# Psychiatric manifestations in HIV infection

**DOI:** 10.1192/j.eurpsy.2022.1218

**Published:** 2022-09-01

**Authors:** T. Jupe, B. Zenelaj

**Affiliations:** 1 Psychiatric Hospital of Attica, 5th Acute Psychiatric Department, Chaidari, Greece; 2 National Center for Children Treatment and Rehabilitationn, Child Psychiatry, Tirana, Albania

**Keywords:** psychiatric symtoms, hiv infection

## Abstract

**Introduction:**

The HIV is a retrovirus, which is immunosuppressive, predisposing the individual to opportunistic infections and certain neoplasm. In addition to impairment in immune functions, evidence has suggested that HIV is neurotropic. It should therefore be anticipated that neuropsychiatric complication might be common in HIV positive individuals during all phases of HIV related illness. The neuropsychiatric aspect of the AIDS remains a challenge for psychiatrists involved in patients care.The relationship between HIV and psychiatric symptoms and conditions is complex and the direction of effects between severe mental illness and HIV infection is unclear. In general, people with severe mental illness are at increased risk of contracting and transmitting HIV, and the prevalence of HIV infection among them is higher than in the general population.

**Objectives:**

To determine how frequently psychiatric symptoms in an HIV positive adult population occur, as well as to determine social, demographic and clinical factors that are associated with the presence of these symptoms.

**Methods:**

Literature review on Pubmed

**Results:**

Depression has a high prevalence in HIV-positive individuals, ranging between 5.8 and 36.0%. Typical features of depression are similar to those in HIVnegative people, although fatigue, loss of appetite and weight, impaired concentration, hopelessness and guilt are more common.Depressed HIV-positive individuals are at high suicide risk.Apathy has also been more commonly reported among HIV patients than in the general population.The prevalence of anxiety among HIV-positive individuals ranges from 4.3 to 44.4%

**Conclusions:**

The rate of psychiatric symptoms in HIV positive patients in this population is high. Most of them go unnoticed and therefore untreated.

**Disclosure:**

No significant relationships.

